# Medication use during pregnancy and pregnancy outcomes in women with immune mediated inflammatory diseases: a UK-based matched cohort study

**DOI:** 10.1093/rheumatology/keag395

**Published:** 2026-08-07

**Authors:** Rachel Charlton, William Tillett, Neil McHugh, Anita McGrogan

**Affiliations:** Department of Life Sciences, University of Bath, Bath, UK; Department of Life Sciences, University of Bath, Bath, UK; Department of Rheumatology, Royal National Hospital for Rheumatic Diseases, Bath, UK; Department of Life Sciences, University of Bath, Bath, UK; Department of Life Sciences, University of Bath, Bath, UK

**Keywords:** pregnancy, psoriatic arthritis, rheumatoid arthritis, psoriasis, spondyloarthritis, inflammatory bowel disease, csDMARDs

## Abstract

**Objectives:**

Immune mediated inflammatory diseases (IMID) affect women of childbearing age, yet there is a lack of evidence about the risk of adverse pregnancy outcomes and the interaction with medication. This study aims to characterize prescribing patterns and pregnancy outcomes in women with an IMID diagnosis.

**Methods:**

Pregnancies to women with rheumatoid arthritis (RA), psoriatic arthritis (PsA), psoriasis (PsO), spondyloarthritis (SpA) and inflammatory bowel disease (IBD) were identified within the Clinical Practice Research Datalink, between 1 January 2000 and 31 June 2020, and matched to pregnancies in women with no such diagnosis. Drug prescribing patterns were described during and surrounding pregnancy. The risk of spontaneous pregnancy loss and major congenital malformations (MCMs) were evaluated compared with women without an IMID. Exposure to commonly prescribed disease-modifying anti-rheumatic drugs was evaluated.

**Results:**

The risk of a spontaneous pregnancy loss ranged from adjusted hazard ratio (HR_adj_) 1.01 (95% CI: 0.79, 1.28) for PsA to HR_adj_ 1.18 (95% CI: 1.07, 1.30) for IBD when compared with women without these conditions. No significant increases in the overall risk of MCMs were observed, with the exception of IBD where it reached borderline significance based on sensitivity analyses [adjusted odds ratio 1.31 (95% CI: 1.00, 1.71)]. No evidence of an increased risk of spontaneous loss was observed in women taking azathioprine, mesalazine and sulfasalazine.

**Conclusion:**

We found no increased risk of spontaneous loss in women with RA, PsA or SpA but a small increase in women with psoriasis or IBD. There was a borderline increased risk of all MCMs in offspring of women with IBD, which should be interpreted cautiously because of potential residual confounding.

Rheumatology key messages

- Medication for immune mediated inflammatory diseases often continues during pregnancy.
- Inflammatory bowel disease may be associated with an increased risk of spontaneous pregnancy loss.
- Azathioprine, mesalazine and sulfasalazine are not associated with an increased risk of spontaneous pregnancy loss.

## Introduction

Rheumatoid arthritis (RA), psoriatic arthritis (PsA), psoriasis (PsO), spondyloarthritis (SpA) and inflammatory bowel disease (IBD) are immune mediated inflammatory diseases (IMID) affecting women of childbearing years. Emerging data from a study looking at fertility indicates that exposure to biologic disease modifying anti-rheumatic drugs (bDMARDs) during conception does not negatively affect conception rates amongst women with RA or PsA and may be associated with improved conception rates amongst women with PsA [1]. However, many conventional synthetic DMARDs (csDMARDs) that are used for the treatment of these conditions are not recommended or are contraindicated in women who are pregnant (or planning pregnancy), either because of their mechanism of action or because of a lack of knowledge about how the drugs may affect the pregnancy and the unborn child [2, 3].

Of the evidence that is available, studies are often small [4–7], from non-UK cohorts [8–14], limited to a narrow range of pregnancy outcomes [15–18] and more recently focus on the newer biologics [1, 19–22] that are often only prescribed in secondary care. Few studies have looked at prescribing patterns of medicines before, during and after pregnancy in women with chronic inflammatory disease [13, 23, 24]. A recent review highlights that women with IMID may be at greater risk of developing adverse pregnancy outcomes but understanding the risk of medications and how they contribute towards adverse pregnancy outcomes needs to be investigated [25]. In addition, the recent British Society for Rheumatology guideline on prescribing drugs in pregnancy and breastfeeding [2] has highlighted the lack of evidence to inform decision making around the time of pregnancy in women with these conditions. More information is needed to help women of childbearing age make decisions about conceiving and management of their disease in relation to pregnancy.

We set out to characterize maternal and infant outcomes and patterns of prescribing amongst women with IMIDs, using data from the United Kingdom’s Clinical Practice Research Datalink (CPRD). The study protocol was approved by a Clinical Practice Research Datalink Expert Review Committee (Protocol number 22_001708).

## Methods

A cohort study was carried out using data from CPRD GOLD, which contains anonymized medical and prescribing data from UK General Practice for ∼15 million patients [26]. All pregnancies to women with an IMID of interest (RA, PsA, PsO, SpA and IBD) were identified in the database between 1 January 2000 and 30 June 2020. Women were required to be aged 14–49 years at the start of pregnancy and have been followed in the CPRD throughout pregnancy and for the one year before and after. The CPRD data includes information on medical symptoms, referrals and diagnoses in the form of Read Clinical codes, and women with an IMID were identified based on diagnosis Read codes and a range of supporting evidence. For patients identified as having more than one IMID, the numbers of Read codes for each condition were reviewed and where there was sufficient evidence to determine that one Read code related to an incorrect working diagnosis, and there were multiple codes for the other, patients were only assigned to the latter. Patients were excluded from the study where it was not possible to determine a single diagnosis, with the exception of patients with both psoriasis and psoriatic arthritis. The start and end dates of each pregnancy were estimated using an algorithm that incorporated information from all pregnancy related codes in the woman’s record [27]. An algorithm was also used to determine the type of pregnancy outcome (live birth, stillbirth and pregnancy loss, which was categorized as spontaneous pregnancy loss, induced loss, ectopic and type of loss unknown). Each pregnancy to a woman with an IMID diagnosis was then matched at a 1:6 ratio to women with no IMID diagnosis, based on the mother’s age, calendar year at pregnancy start and GP practice. For pregnancies ending in a live birth, the medical records of the mothers were linked to those of the child, where possible, using an algorithm described previously [27].

All non-steroidal anti-inflammatory drug (NSAID), corticosteroid and csDMARD prescriptions issued to women with an IMID in the year before, during and year after pregnancy were identified. Major congenital malformations (MCMs) were identified in the medical records of the infants of mother–baby pairs based on Read codes recorded in the first year of life and defined according to the EUROCAT classification [28]. For Read codes that were less specific (e.g. ‘congenital malformation’) or where a malformation could be major or minor, depending on specific details (e.g. hydronephrosis, which is considered minor if the pelvis dilation is <10 mm, and clubfoot, which is minor if it is of a postural in origin), the MCM was classed as a ‘possible MCM’ if the supporting evidence to confirm that it met the major criteria was not available in the record. Covariate information was extracted on maternal age at the start of pregnancy, maternal smoking status, body-mass-index at the start of pregnancy, as well as pre-pregnancy diagnoses of epilepsy, diabetes, hypertension, hyperthyroidism, hypothyroidism, sickle cell anaemia and thalassemia.

### Analysis

The patient characteristics for all women with a pregnancy were reported stratified by IMID and for the non-IMID matched comparators. Chi-squared tests were used to make comparisons between groups. For patients with psoriasis, all analyses were reported restricted to those patients who did not have a known diagnosis of PsA. Patterns of NSAID, corticosteroid and csDMARD prescribing were described in 3-month periods in the year leading up to pregnancy, each pregnancy trimester and the year following pregnancy. Exposure was based on the issue of ≥1 prescription during the time period of interest.

The absolute risk of each of the pregnancy outcomes of interest was calculated for women with each IMID and for the matched pregnancies to women without an IMID. For spontaneous pregnancy losses, Cox proportional hazard models with left truncation at 8 weeks were used to account for the unknown number of pregnancies where a loss occurs before the pregnancy is recognized, as has been used in previous research [29]. Pregnancies were excluded where the woman had received ≥1 prescription for a known non-csDMARD teratogen during the first trimester of pregnancy. Where variables were found to violate the proportional hazard assumption, the models were run with those variables as separate strata. A sensitivity analysis was carried out including those pregnancy losses where the type of loss was unknown as a spontaneous loss. Adjustments were made for confounding factors where appropriate based on what has been previously reported in the literature. For the csDMARDs with sufficient sample size (azathioprine, mesalazine and sulfasalazine) the risk of a spontaneous pregnancy loss was calculated for women with first trimester monotherapy exposure to the product and no NSAID or corticosteroid co-prescribing, compared with women with an IMID and no exposure during the first trimester to any csDMARD, NSAID or corticosteroid.

For MCMs, infants diagnosed with an MCM in addition to a syndrome were excluded as these were unlikely to be drug induced. For the remainder, the absolute risk of MCMs was calculated stratified by IMID. Logistic regression was used to determine the likelihood of an MCM in the offspring of women with each IMID compared with women without. Adjustments were made for confounding factors where appropriate. All analyses were carried out using Stata (version 16.1; Stata Corp., College Station, TX, USA).

## Results

In total, 22 081 women were identified with an IMID diagnosis before pregnancy and had 34 367 pregnancies during the study period. An additional 844 women were identified with an IMID diagnosis during their pregnancy. Table 1 shows the patient characteristics for women with pregnancies following an IMID diagnosis and the matched non-IMID comparator pregnancies. Supplementary Table S1 gives a breakdown of IMID diagnoses during pregnancy by trimester. The mean age at the start of pregnancy ranged from 30.2 years for patients with PsO to 32.8 years for SpA. The mean age at IMID diagnosis was younger for patients with PsO (20.9 years) and older for patients with PsA (27.2 years), with PsA patients having the shortest mean disease duration at the start of pregnancy and PsO having the longest (5.7 and 9.3 years, respectively). A larger percentage of PsO patients were smokers compared with the other IMIDs and a larger percentage of PsA patients were categorized as obese. Autoimmune thyroid disease was more common in patients with RA (5.8%). A larger percentage of RA and PsA patients received a prescription for a known teratogen during pregnancy (4.4 and 4.3%, respectively).

**Table 1:** Patient characteristics for the different immune mediated inflammatory diseases (IMID) and matched non-IMID comparators.

Characteristic	RA	No RA	PsA	No PsA	PsO	No PsO	SpA	No SpA	IBD	No IBD
	(*n* = 1311)	(*n* = 7866)	(*n* = 573)	(*n* = 3438)	(*n* = 27 188)	(*n* = 163 127)	(*n* = 493)	(*n* = 2958)	(*n* = 4802)	(*n* = 28 812)
Maternal age, mean (s.d.), years	32.5 (5.8)	32.5 (5.7)	32.4 (5.7)	32.3 (5.6)	30.2 (6.2)	30.2 (6.2)	32.8 (5.4)	32.8 (5.4)	31.7 (5.7)	31.7 (5.6)
IMID diagnosis age, mean (s.d.), years	24.6 (9.5)	—	27.2 (6.8)	—	20.9 (8.4)	—	26.3 (7.1)	—	25.0 (6.8)	—
Disease duration, mean (s.d.), years	8.4 (7.9)	—	5.7 (5.0)	—	9.3 (7.5)	—	7.0 (5.9)	—	7.2 (5.7)	—
Smoking status, *n* (%)										
Smoker	300 (22.9)	2021 (25.7)	129 (22.5)	882 (25.7)	9811 (36.1)	48 411 (29.7)	140 (28.4)	735 (24.8)	1067 (22.2)	7431 (25.8)
Ex-smoker	308 (23.5)	1646 (20.9)	147 (25.7)	752 (21.9)	5819 (21.4)	31 224 (19.1)	108 (21.9)	654 (22.1)	1256 (26.2)	5970 (20.7)
Non-smoker	701 (53.5)	4177 (53.1)	297 (51.8)	1798 (52.3)	11 467 (42.2)	82 951 (50.9)	243 (49.3)	1565 (52.9)	2470 (51.4)	15 325 (53.2)
Unknown	≤5	22 (0.3)	0 (0.0)	6 (0.2)	91 (0.3)	541 (0.3)	≤5	≤5	9 (0.2)	86 (0.3)
Alcohol drinking, *n* (%)										
Drinker	898 (68.5)	5482 (69.7)	396 (69.1)	2379 (69.2)	18 033 (66.3)	108 266 (66.4)	351 (71.2)	2105 (71.2)	3313 (69.5)	19 811 (69.2)
Ex-drinker	80 (6.1)	428 (5.4)	47 (8.2)	160 (4.7)	1590 (5.8)	8076 (5.0)	21 (4.3)	157 (5.3)	295 (6.2)	1407 (4.9)
Heavy drinker*	9 (0.7)	100 (1.3)	≤5	51 (1.5)	583 (2.1)	2883 (1.8)	0 (0.0)	43 (1.5)	58 (1.2)	463 (1.6)
Non-drinker	215 (16.4)	968 (12.3)	68 (11.9)	407 (11.8)	3313 (12.2)	20 038 (12.3)	76 (15.4)	341 (11.5)	622 (13.0)	3454 (12.1)
Unknown	109 (8.3)	888 (11.3)	58 (10.1)	441 (12.8)	3669 (13.5)	23 864 (14.6)	45 (9.1)	312 (10.5)	482 (10.1)	3485 (12.2)
Body mass index, *n* (%)										
<18.5	37 (2.8)	123 (1.6)	10 (1.7)	45 (1.3)	432 (1.6)	2996 (1.8)	8 (1.6)	41 (1.4)	150 (3.1)	415 (1.4)
18.5–24.9	387 (29.5)	2024 (25.7)	124 (21.6)	796 (23.2)	6953 (25.6)	41 429 (25.4)	148 (30.0)	770 (26.0)	1568 (32.7)	7134 (24.8)
25.0–29.9	189 (14.4)	1145 (14.5)	78 (13.6)	471 (13.7)	4098 (15.1)	23 600 (14.5)	76 (15.4)	437 (14.8)	731 (15.2)	4268 (14.8)
≥30.0	179 (13.7)	1018 (12.9)	101 (17.6)	414 (12.0)	3890 (14.3)	20 375 (12.5)	50 (10.1)	334 (11.3)	463 (9.6)	3461 (12.0)
Unknown	519 (39.6)	3556 (45.2)	260 (45.4)	1712 (49.8)	11 815 (43.5)	74 727 (45.8)	211 (42.8)	1376 (46.5)	1890 (39.4)	13 534 (47.0)
Diabetes, *n* (%)										
Type 1	61 (4.7)	206 (2.6)	33 (5.5)	88 (2.5)	651 (2.4)	3393 (2.1)	19 (3.9)	74 (2.5)	130 (2.7)	671 (2.3)
Gestational	31 (2.4)	148 (1.9)	11 (1.8)	86 (2.4)	643 (2.4)	3010 (1.8)	13 (2.6)	76 (2.6)	94 (2.0)	535 (1.9)
Epilepsy, *n* (%)	22 (1.7)	105 (1.3)	7 (1.2)	56 (1.6)	421 (1.5)	2426 (1.5)	≤5	44 (1.5)	90 (1.9)	392 (1.4)
Hyperthyroidism, *n* (%)	26 (2.0)	76 (1.0)	≤5	26 (0.7)	171 (0.6)	1057 (0.6)	≤5	35 (1.2)	25 (0.5)	266 (0.9)
Hypothyroidism, *n* (%)	76 (5.8)	199 (2.5)	11 (1.8)	98 (2.7)	644 (2.4)	3161 (1.9)	11 (2.2)	72 (2.4)	93 (1.9)	678 (2.4)
Antihypertensive Rx, *n* (%)	133 (10.1)	933 (11.9)	72 (12.6)	471 (13.7)	3361 (12.4)	19 330 (11.8)	61 (12.4)	368 (12.4)	563 (11.7)	3536 (12.3)
Teratogen Rx, *n* (%)	56 (4.3)	44 (0.6)	25 (4.4)	15 (0.4)	107 (0.4)	707 (0.4)	≤5	12 (0.4)	104 (2.1)	123 (0.4)

IBD: inflammatory bowel disease; PsA: psoriatic arthritis; PsO: psoriasis; RA: rheumatoid arthritis; SpA: spondyloarthritis; teratogen Rx: issue of a prescription for a known teratogen during pregnancy. *Heavy drinker was based on a code for heavy or problem drinking or evidence of >42 units per week for males and >31 units per week for females.

**Figure 1:**
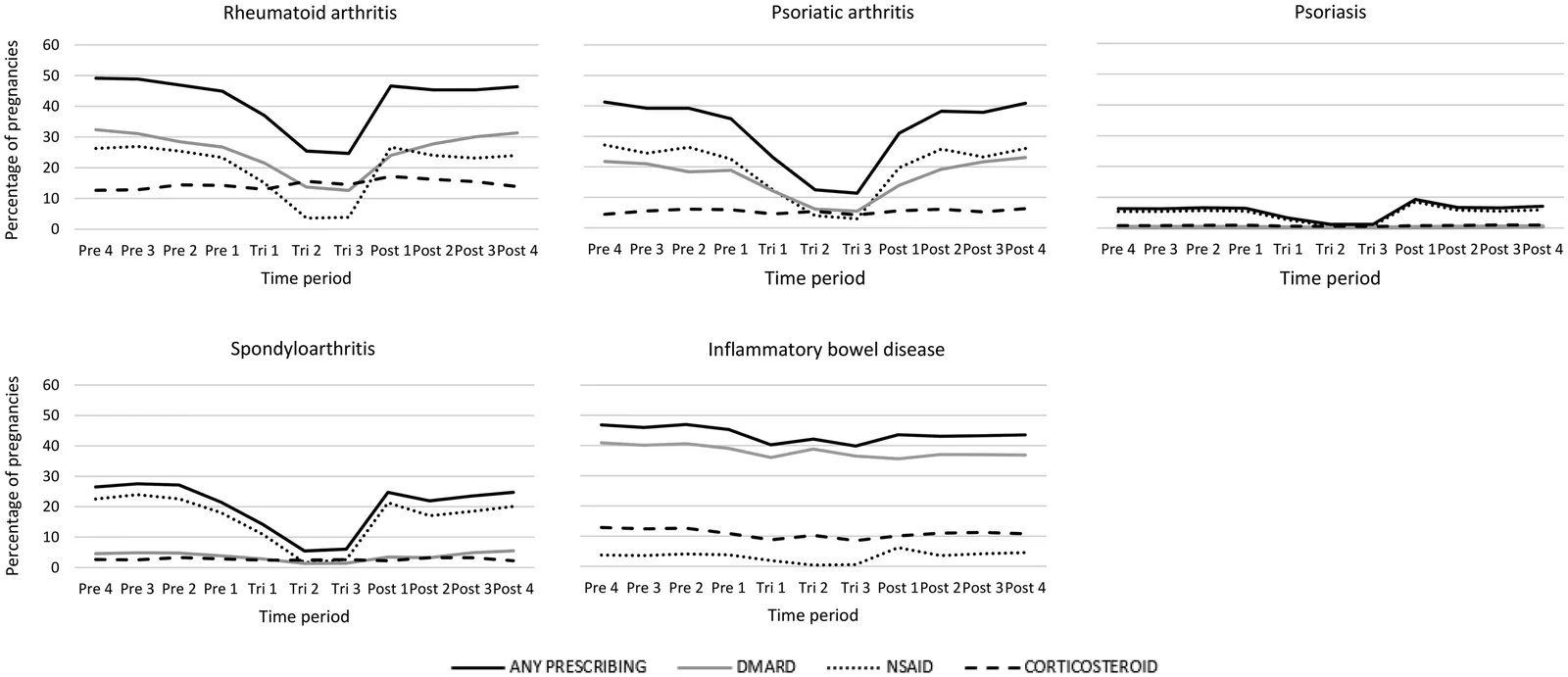
Percentages are shown separately for each IMID and each 3-month time period in the year before, during and year after pregnancy. The solid black line shows the percentage of women receiving any prescribing and the grey and dashed lines show the percentage receiving a prescription for each of the drug classes. The percentages shown for each of the drug classes will sum to more than the any prescribing percentage as some women will have received a prescription for more than one drug class. DMARD: disease modifying anti-rheumatic drug; NSAID: non-steroidal anti-inflammatory drug; tri: trimester

In the year before pregnancy, between 18.6% (PsO) and 68.8% (RA) of women with a IMID diagnosis received ≥1 prescription for an NSAID, corticosteroid or csDMARD; this reduced to between 4.5% (PsO) and 48.9% (IBD) during pregnancy (Supplementary Fig. S1). Figure 1 shows the prescribing by drug class in 3-monthly time periods before, during and after pregnancy. With the exception of IBD, there was a clear decline in overall prescribing, starting 3–6 months before the start of pregnancy and returning to close to pre-pregnancy levels after pregnancy. This was observed for csDMARDs and NSAIDs but not corticosteroids. Women with IBD were more likely to continue their csDMARD prescribing throughout pregnancy. Supplementary Figure S2 shows the breakdown of csDMARD prescribing by product. Sulfasalazine, hydroxychloroquine and methotrexate were the most commonly prescribed for RA, PsA and SpA whilst mesalazine and azathioprine were most commonly prescribed for IBD. Of those receiving a csDMARD prescription during pregnancy (*n* = 2504), 85.1% received a prescription during the first trimester with only 42.5% receiving one in all three trimesters, of which 89.4% of these were women with IBD. For women receiving prescriptions for methotrexate (*n* = 68) and leflunomide (*n* = 13) during pregnancy, which are known to be teratogenic, 80.9% and 84.6% only received a prescription during the first trimester.

Table 2 shows a breakdown of the type of pregnancy outcome for each IMID and the non- IMID matched pregnancies. Between 68.6% (RA) and 70.4% (IBD) of pregnancies ended in a live birth compared with 69.4% and 71.0% in the matched comparator groups respectively. Table 3 shows the results of the Cox proportional hazards models of each individual IMID. After adjustment, women with IBD were found to have an increased risk of a spontaneous pregnancy loss [adjusted hazard ratio (HR_adj_) 1.18 (95% CI: 1.07, 1.30)], and this increase in risk remained following the inclusion of those pregnancies where the type of loss (spontaneous or induced) was unknown [HR_adj_ 1.16 (95% CI: 1.07, 1.26)] (Supplementary Table S2). A small increase in risk was also observed for women with psoriasis [HR_adj_ 1.05 (95% CI: 1.02, 1.09), and including the unknowns HR_adj_ 1.02 (95% CI: 0.99, 1.05)].

**Table 2:** Pregnancy outcomes for women with an immune mediated inflammatory disease (IMID) diagnosis before the start of pregnancy and the matched non-IMID comparators.

Outcome	RA	No RA	PsA	No PsA	PsO	No PsO	SpA	No SpA	IBD	No IBD
	(*n* = 1311)	(*n* = 7866)	(*n* = 573)	(*n* = 3438)	(*n* = 27 188)	(*n* = 163 127)	(*n* = 493)	*n* = 2958)	(*n* = 4802)	(*n* = 28 812)
Livebirth, *n* (%)	899 (68.6)	5458 (69.4)	403 (70.3)	2399 (69.8)	18 900 (69.5)	114 886 (70.4)	346 (70.2)	2047 (69.2)	3360 (70.4)	20 460 (71.0)
Stillbirth, *n* (%)	7 (0.5)	30 (0.4)	≤5	21 (0.6)	101 (0.4)	682 (0.4)	≤5	17 (0.6)	24 (0.5)	112 (0.4)
Pregnancy loss, *n* (%)	405 (30.9)	2378 (30.2)	167 (29.1)	1018 (29.6)	8187 (30.1)	47 559 (29.2)	144 (29.2)	894 (30.2)	1418 (29.7)	8240 (28.6)
Spontaneous	222 (16.9)	1231 (15.6)	103 (18.0)	537 (15.6)	4026 (14.8)	22 441 (13.8)	89 (18.1)	468 (15.8)	793 (16.5)	4211 (14.6)
Ectopic	16 (1.2)	86 (1.1)	7 (1.2)	52 (1.5)	305 (1.1)	1665 (1.0)	6 (1.2)	24 (0.8)	58 (1.2)	302 (1.0)
Induced	95 (7.2)	626 (8.0)	31 (5.4)	246 (7.2)	2348 (8.6)	14 279 (8.8)	33 (6.7)	251 (8.5)	318 (6.6)	2206 (7.7)
Unknown	72 (5.5)	435 (5.5)	26 (4.5)	183 (5.3)	1508 (5.5)	9174 (5.6)	16 (3.2)	151 (5.1)	249 (5.2)	1521 (5.3)

IBD: inflammatory bowel disease; PsA: psoriatic arthritis; PsO: psoriasis; RA: rheumatoid arthritis; SpA: spondyloarthritis.

**Table 3:** Risk of spontaneous pregnancy loss in women with each immune mediated inflammatory disease (IMID) compared with those in the matched non-IMID comparators.

IMID	Unadjusted HR (95% CI)	*P*	Adjusted HR (95% CI)^a^	*P*
Rheumatoid arthritis	1.09 (0.94, 1.27)	0.240	1.06 (0.90, 1.27)	0.480
Psoriatic arthritis	1.11 (0.89, 1.38)	0.352	1.01 (0.79, 1.28)	0.948
Psoriasis	1.07 (1.03, 1.11)	0.000	1.05 (1.02, 1.09)	0.004
Spondyloarthritis	1.12 (0.89, 1.41)	0.351	1.08 (0.86, 1.38)	0.497
Inflammatory bowel disease	1.12 (1.04, 1.21)	0.005	1.18 (1.07, 1.30)	0.001

a Adjusted for maternal age, smoking status, body-mass-index, treated hypertension, diabetes before pregnancy, first trimester prescribing of non-teratogenic conventional synthetic disease modifying anti-rheumatic drugs (csDMARD), teratogenic csDMARDs, corticosteroids and non-steroidal anti-inflammatory drugs. HR: hazard ratio.

Supplementary Table S3 shows the results of the Cox proportional hazards models looking at the risk of a spontaneous pregnancy loss in women with an IMID following first trimester monotherapy exposure to azathioprine, mesalazine or sulfasalazine in the absence of NSAID and corticosteroid exposure, compared with women with an IMID and no exposure to any csDMARD, NSAID or corticosteroid. For all three products, no significant increase in risk was observed [HR_adj_ 1.16 (95% CI: 0.80, 1.68), HR_adj_ 0.87 (95% CI: 0.67, 1.13) and HR_adj_ 0.73 (95% CI: 0.42, 1.28) for azathioprine (*n* = 272), mesalazine (*n* = 873) and sulfasalazine (*n* = 132), respectively]. Sensitivity analyses, including those pregnancy losses where the type of loss was unknown, did not change the findings.

Of the 23 908 IMID pregnancies and 145 250 non-IMID pregnancies ending in a live birth, it was possible to link the mother’s medical record to the medical record of the infant(s) for 19 601 (82.0%) and 118 270 (81.4%), respectively, who were still contributing to the CPRD at 1 year of age or had died before reaching one year of age. Supplementary Table S4 shows the percentage of linked pregnancies for which evidence of a MCM was identified, stratified by IMID, irrespective of prescribing. The absolute risk of an MCM ranged from 1.2% in those with RA to 2.1% in those with IBD, increasing to 1.4% and 2.5%, respectively, when including those cases of possible MCMs. When compared with the non-IMID pregnancies and adjusting for potential confounders where possible, there was no evidence to suggest an increased risk of an MCM in pregnancies to women with an IMID (Table 4). Including those MCMs identified as ‘possible’, MCMs did not substantially alter the adjusted odds ratios (OR_adj_) for all the IMIDs, with the exception of IBD. For women with IBD the risk increased to OR_adj_ 1.31 (95% CI: 1.00, 1.71) and was borderline significant (*P* = 0.053) (Supplementary Table S5). Small numbers prevented it from being possible to look at the risk associated with first trimester prescribing.

**Table 4:** Risk of a major congenital malformation in pregnancies to women with an immune mediated inflammatory disease (IMID) compared with those in the matched non-IMID comparators.

IMID	Unadjusted OR (95% CI)	*P*	Adjusted OR (95% CI)	*P*
Rheumatoid arthritis	0.78 (0.39, 1.58)	0.492	0.76^a^ (0.37, 1.56)	0.460
Psoriatic arthritis	0.95 (0.40, 2.26)	0.903	0.87^a^ (0.36, 2.10)	0.750
Psoriasis	1.00 (0.87, 1.13)	0.944	0.99^b^ (0.87, 1.13)	0.925
Spondyloarthritis	1.01 (0.39, 2.63)	0.981	1.01^c^ (0.39, 2.62)	0.989
Inflammatory bowel disease	1.21 (0.91, 1.62)	0.191	1.20^a^ (0.89, 1.60)	0.232

a Adjusted for maternal age, smoking status and high dose folic acid prescribing.

b Adjusted for maternal age, smoking status, high dose folic acid prescribing, treated hypertension and diabetes before pregnancy.

c Adjusted for maternal age and smoking status. OR: odds ratio.

## Discussion

This study has demonstrated that although many women with IMIDs discontinue treatment prior to pregnancy a substantial proportion do continue to receive prescriptions, particularly those with a diagnosis of IBD. The study identified evidence of a small increase in risk of spontaneous pregnancy loss in women with psoriasis or IBD. It also identified a possible increase in risk of MCMs in women with IBD. For RA, PsA and SpA no evidence of increases in risk was observed. This study, however, primarily assessed associations rather than the causal effects of medication. The associations warrant cautious interpretation and require validation in other datasets, due to the potential for residual confounding and a lack of information on the impact of disease activity that likely influences both treatment continuation and pregnancy outcomes.

The difference in treatment discontinuation between the specialities is not surprising, as a flare of IBD can cause life threatening complications whilst in general many of the other conditions, for some women, tend to ameliorate during pregnancy [30]. Despite guidelines advising that methotrexate and leflunomide should be avoided in pregnancy and discontinued prior to planned conception [2], this study identified a number of women who received a prescription for these products during pregnancy. This may reflect that these pregnancies were unplanned and highlights the need for pregnancy planning and continuous education and preconception counselling to minimize inadvertent pregnancy exposure.

Our study identified small increases in risk of spontaneous pregnancy loss for women with psoriasis and women with IBD. However, these warrant cautious interpretation given the lack of information on disease activity, which is an important unmeasured confounder. To date, studies looking at adverse pregnancy outcomes in psoriasis have been conflicting, with some demonstrating a slight increase in risk of pregnancy loss, particularly in women with moderate to severe disease severity [31], and others finding no evidence of an increase in risk [14, 32]. For IBD, a Korean study has reported an increased risk of spontaneous pregnancy loss for moderate to severe IBD [OR 1.33 (95% CI: 1.04, 1.68)] but not for mild disease [33]. A meta-analysis comparing active *vs* inactive IBD reported an odds ratio of 1.87 (95% CI: 1.17, 3.0) for spontaneous pregnancy loss [33] and highlighted the importance of continuous disease control even during pregnancy.

In our study, we applied left truncation at 8 weeks to account for the unknown number of pregnancies where a loss occurred before the pregnancy was recognized. However, as a large percentage of spontaneous pregnancy losses occur within the first 8 weeks of gestation, it is possible that by excluding this critical window the analysis may selectively include only pregnancies that have already ‘survived’ the highest-risk period. If IMIDs are associated with earlier spontaneous loss then the inability to capture these could have underestimated the true burden of spontaneous loss in the IMID population and obscured important differences.

The finding of no evidence of an increased risk of spontaneous pregnancy loss in women with an IMID exposed to azathioprine, mesalazine and sulfasalazine during the first trimester of pregnancy is reassuring and supports current clinical guidelines that these products are generally thought to be safe to use during pregnancy [2].

With the exception of IBD, our study did not find evidence to suggest an increased risk of MCMs in the offspring of women with any of the IMIDs when compared with those without. This is largely in line with other studies, including a meta-analysis looking at psoriatic disease that found no statistically increased risks of neonatal adverse outcomes [34]. One meta-analysis has reported an increase in risk of MCMs for women with IBD [OR 1.29 (95% CI: 1.05, 1.58)] but reported that it may be unreliable, secondary to publication bias [35]. A second meta-analysis reported a pooled odds ratio for congenital anomalies of 3.03 (95% CI: 1.43, 6.42) comparing patients with Crohn’s disease compared with healthy controls [36]. In our study, a significant increase in risk of MCMs was only observed for women with IBD in the sensitivity analysis including the ‘possible’ MCMs. It is likely that only some of the 11 additional cases included will be true MCMs and this may therefore be an overestimate. Our study, however, was not able to identify MCMs that were prenatally diagnosed where the pregnancy was then subsequently terminated. If women with IMIDs are at a higher risk for MCMs where a prenatal diagnosis would warrant an elective termination of the pregnancy, then this could have potentially underestimated the true prevalence of MCMs in the IMID population. A number of studies have reported on additional adverse pregnancy outcomes that were not included in this study; these include caesarean section, pre-term birth and postpartum depression. Some of these studies have reported evidence of, or suggested, an increased risk of caesarean section in women with a diagnosis of IBD, psoriatic disease, SpA and RA [8, 9, 11, 25, 37–39], a higher rate of preterm birth in psoriatic disease, SpA and RA [8, 25, 34, 37–39], and a higher rate of postpartum depression in women with rheumatic disease compared with those without [40]. One study, looking at disease severity in RA, found that increasing disease severity was associated with an increased risk for preterm delivery and small for gestational age and concluded that it might be possible to lower the risks if RA is well controlled early in pregnancy [15].

This study had a number of strengths including its size, duration of follow-up, inclusion of pregnancies that ended in a pregnancy loss and the representative nature of the study population and subsequent generalizability. There were, however, a number of limitations, including the fact that the start of pregnancy was algorithmically estimated meaning that in some cases the start date of the pregnancy used for determining exposure status may have been incorrectly estimated and in some cases may have led to misclassification of exposure. Prescribing data were limited to prescriptions issued by a GP in primary care and were based on the issue of ≥1 prescription. There was no information on adherence and it was not possible to know whether the medication was actually taken at the dose and for the duration instruction, and this could have led to exposure misclassification, particularly for short and incidental exposures. Although the nature of the healthcare system in the UK means it is likely that the majority of NSAID, corticosteroid and csDMARD prescribing was captured, it was not possible to capture prescriptions issued in secondary care or NSAIDs purchased over-the-counter without a prescription. It was also not possible to capture the prescribing of biologics, which will have had a greater impact in the latter years of the study and in women with more severe disease. Whilst every attempt was made to avoid misclassification of IMIDs and women were excluded where the precise diagnosis was uncertain or multiple IMIDs may have been present, the study did not consider the presence of connective tissue diseases. It is therefore possible that some women may have been misclassified or had a coexisting condition such as systemic lupus erythematosus, which is known to be associated with adverse pregnancy outcomes [41]. Although our study attempted to take into account a number of potential confounders, there may still have been confounding by indication and it did not look at measures of disease activity. Disease activity was therefore an unmeasured confounder and is known to influence pregnancy outcomes in patients with IBD and psoriasis as well as being a key determinant in treatment decisions. In future studies it would be interesting to see if prescribing persistence could be used as a proxy for disease severity. The study also did not look at parity/pregnancy history or socioeconomic status.

For some pregnancy losses it was not possible to determine whether the loss was spontaneous or induced. It is likely that these pregnancies comprised a mixture of both types of loss and that the analyses where these were excluded will have underestimated the absolute risk of spontaneous pregnancy loss. There will also have been some women who had a spontaneous pregnancy loss before the pregnancy was recognized, and hence delaying study entry until the ninth week of gestation will have helped to prevent the introduction of any selection bias, but if women with an IMID are at a greater risk of earlier losses then this would not be the case. This study did not look at fertility in women with IMIDs. When looking at MCMs, the nature of the data meant that the study was limited to pregnancies that ended in a live birth and any MCMs that were identified prenatally that resulted in the pregnancy being terminated were not captured. In addition, some MCMs will be diagnosed later in life and will not have been captured as a result of the first year of life cut-off used in this study. Any diagnoses carried out via private healthcare or where the infant received only hospital care may also not have been captured. The prevalences of MCMs in the livebirths were, however, in line with those reported in the 2020 Congenital Anomaly Official Statistics Report [42]. The limitations of this study and gaps in data availability and accuracy emphasize the need and importance of well controlled, organized data collection in real life or prospective studies, to complement and enhance the retrospective study designs, data availability and methodologies.

In this study we have characterized maternal and infant outcomes and patterns of prescribing amongst women with IMIDs in a large primary care cohort. We found that many women stopped medication prior to pregnancy. We found no evidence to suggest there was an increased risk of spontaneous pregnancy loss in women with an RA, PsA or SpA diagnosis compared with those without but did find evidence of a small increase in women with psoriasis or IBD. IBD was the only IMID where evidence was found to potentially suggest an increased risk of MCMs. These findings are hypothesis generating and warrant cautious interpretation due to the potential for residual confounding and a lack of information on the impact of disease activity, which likely influences both treatment continuation and pregnancy outcomes. No evidence was found in this observational study to suggest an increased risk of spontaneous loss in women taking azathioprine, mesalazine and sulfasalazine. The prescribing data in this study add to the existing literature and can be used to help support women with IMIDs and healthcare professionals make informed decisions when planning pregnancy. It also highlights the need for pregnancy planning and continuous education and counselling to women with IMIDs during their childbearing years.

## Supplementary Material

keag395_Supplementary_Data

## Data Availability

This study is based in part on data from the Clinical Practice Research Datalink obtained under licence from the UK Medicines and Healthcare products Regulatory Agency. The data are provided by patients and collected by the NHS as part of their care and support. The interpretation and conclusions contained in this study are those of the authors alone.
