# Supplementary material for: Medication use during pregnancy and pregnancy outcomes in women with immune mediated inflammatory diseases: a UK-based matched cohort study
Source: Rheumatology (Oxford). 2026 Aug 7;65(8):keag395. doi: 10.1093/rheumatology/keag395 (PMC13499226; doi:10.1093/rheumatology/keag395)
Supplement: Supplementary file 1 [file keag395_supplementary_data.docx]

**Online Supplementary Material**

Medication use during pregnancy and pregnancy outcomes in women with immune mediated inflammatory diseases:

a UK-based matched cohort study

**Supplementary table S1** Number of pregnancies where the woman was diagnosed with an immune mediated inflammatory disease before and during the pregnancy

|  | | **Time of diagnosis** | | | | | | | |
| --- | --- | --- | --- | --- | --- | --- | --- | --- | --- |
|  | **Total** | **Before pregnancy** | | **Trimester 1** | | **Trimester 2** | | **Trimester 3** | |
| **Rheumatoid arthritis** | 1,356 | 1,311 | (96.7) | 33 | (2.4) | 7 | (0.5) | ≤5 | NA |
| **Psoriatic arthritis** | 591 | 573 | (97.0) | 10 | (1.7) | 6 | (1.0) | ≤5 | NA |
| **Psoriasis** | 27,657 | 27,188 | (98.3) | 281 | (1.0) | 112 | (0.4) | 76 | (0.3) |
| **Spondyloarthritis** | 509 | 493 | (96.9) | 9 | (1.8) | ≤5 | NA | ≤5 | NA) |
| **Inflammatory bowel disease** | 4,981 | 4,802 | (96.4) | 72 | (1.4) | 67 | (1.3) | 40 | (0.8) |

**Supplementary table S2** Sensitivity analysis of the risk of spontaneous pregnancy loss in women with immune mediate inflammatory (IMID) disease compared to the non-IMID comparators, including those where the type of loss was unknown

| **Immune mediated inflammatory disease** |  | | | | | |
| --- | --- | --- | --- | --- | --- | --- |
|  | **Unadjusted** | | | **Adjusted^†^** | | |
|  | **Hazard ratio** | **CI_95_** | ***P*** | **Hazard ratio** | **CI_95_** | ***P*** |
| **Rheumatoid arthritis** | 1.06 | 0.94-1.21 | 0.341 | 1.03 | 0.89-1.20 | 0.655 |
| **Psoriatic arthritis** | 1.02 | 0.84-1.24 | 0.812 | 0.95 | 0.76-1.17 | 0.610 |
| **Psoriasis** | 1.04 | 1.01-1.08 | 0.003 | 1.02 | 0.99-1.05 | 0.112 |
| **Spondyloarthritis** | 1.00 | 0.81-1.24 | 0.996 | 0.96 | 0.77-1.19 | 0.686 |
| **Inflammatory bowel disease** | 1.08 | 1.01-1.16 | 0.025 | 1.16 | 1.07-1.26 | 0.001 |

**^†^**adjusted for maternal age, smoking status, body-mass-index, treated hypertension, diabetes before pregnancy, first trimester prescribing of non-teratogenic conventional synthetic disease modifying anti-rheumatic drugs (csDMARD), teratogenic csDMARDs, corticosteroids and non-steroidal anti-inflammatory drugs

**Supplementary table S3** Risk of spontaneous pregnancy loss in women with an IMID and monotherapy exposure during the first trimester in the absence of NSAIDs and corticosteroids compared to women with an IMID and no csDMARD, NSAID or corticosteroid exposure

| **First trimester exposure** |  | | | | | |
| --- | --- | --- | --- | --- | --- | --- |
|  | **Unadjusted** | | | **Adjusted^†^** | | |
|  | **Hazard ratio** | **CI_95_** | ***P*** | **Hazard ratio** | **CI_95_** | ***P*** |
| **Azathioprine** | 1.18 | (0.88-1.59) | 0.270 | 1.16 | (0.80-1.68) | 0.433 |
| **Mesalazine** | 0.99 | (0.83-1.18) | 0.902 | 0.87 | (0.67-1.13) | 0.299 |
| **Sulfasalazine** | 1.02 | (0.65-1.60) | 0.929 | 0.73 | (0.42-1.28) | 0.272 |
|  |  |  |  |  |  |  |

^†^adjusted for maternal age, smoking status, body-mass-index, treated hypertension, diabetes before pregnancy and stratified by immune mediated inflammatory disease

**Supplementary table S4** Major congenital malformations in women with an immune mediated inflammatory disease diagnosis before the start of pregnancy and their matched non-IMID comparators, regardless of type of prescribing

|  | **RA** | | **No RA** | | **PsA** | | **No PsA** | | **PsO** | | **No PsO** | | **SpA** | | **No SpA** | | **IBD** | | **No IBD** | |
| --- | --- | --- | --- | --- | --- | --- | --- | --- | --- | --- | --- | --- | --- | --- | --- | --- | --- | --- | --- | --- |
|  | **N** | **(%)** | **N** | **(%)** | **N** | **(%)** | **N** | **(%)** | **N** | **(%)** | **N** | **(%)** | **N** | **(%)** | **N** | **(%)** | **N** | **(%)** | **N** | **(%)** |
|  | **761** |  | **4515** |  | **317** |  | **1854** |  | **15323** |  | **92598** |  | **288** |  | **1748** |  | **2706** |  | **16590** |  |
| **Definite MCM** | 9 | (1.2) | 68 | (1.5) | 6 | (1.9) | 37 | (2.0) | 267 | (1.7) | 1621 | (1.8) | 5 | (1.7) | 30 | (1.7) | 56 | (2.1) | 284 | (1.7) |
| **Including possible MCM^*^** | 11 | (1.4) | 82 | (1.8) | 7 | (2.2) | 38 | (2.0) | 320 | (2.1) | 1883 | (2.0) | 7 | (2.4) | 34 | (1.9) | 67 | (2.5) | 313 | (1.9) |

RA = Rheumatoid arthritis, PsA = Psoriatic arthritis, PsO = Psoriasis, SpA = Spondyloarthritis, IBD = Inflammatory Bowel Disease, MCM = major congenital malformation

^*^ Possible MCMs include those where the Read codes were less specific (e.g. “congenital malformation”) or where a malformation could be major or minor, depending on specific details (e.g. hydronephrosis which is considered minor if the pelvis dilation is <10mm and clubfoot which is minor if it is of a postural in origin), and the supporting evidence to confirm that it met the major criteria was not available in the record.

**Supplementary table S5** Risk of a major congenital malformation in pregnancies to women with an immune mediated inflammatory disease (IMID) compared to pregnancies without, including possible MCMs

| **IMID** |  | | | | | |
| --- | --- | --- | --- | --- | --- | --- |
|  | **Unadjusted** | | | **Adjusted** | | |
|  | **Odds ratio** | **CI_95_** | ***P*** | **Odds ratio** | **CI_95_** | ***P*** |
| **Rheumatoid arthritis** | 0.79 | 0.42-1.49 | 0.473 | 0.78^†^ | 0.41-1.49 | 0.449 |
| **Psoriatic arthritis** | 1.08 | 0.48-2.44 | 0.855 | 1.00^†^ | 0.44-2.29 | 0.999 |
| **Psoriasis** | 1.03 | 0.91-1.16 | 0.657 | 1.03^‡^ | 0.91-1.16 | 0.661 |
| **Spondyloarthritis** | 1.26 | 0.55-2.86 | 0.588 | 1.25^±^ | 0.55-2.86 | 0.592 |
| **Inflammatory bowel disease** | 1.32 | 1.01-1.72 | 0.041 | 1.31^†^ | 1.00-1.71 | 0.053 |

^†^ adjusted for maternal age, smoking status and high dose folic acid prescribing; ^‡^ adjusted for maternal age, smoking status, high dose folic acid prescribing, treated hypertension and diabetes before pregnancy; ^±^ adjusted for maternal age and smoking status

Percentage of pregnancies

| Immune mediated inflammatory disease | Year pre-pregnancy | During pregnancy | Year post-pregnancy |
| --- | --- | --- | --- |
| RA | 68.8024 | 41.5713 | 75.4386 |
| PsA | 63.1763 | 26.5271 | 70.1571 |
| PsO | 18.6374 | 4.48632 | 32.7668 |
| SpA | 50.1014 | 16.8357 | 53.9554 |
| IBD | 66.0766 | 48.6464 | 73.1154 |

RA = Rheumatoid arthritis, PsA = Psoriatic arthritis, PsO = Psoriasis, SpA = Spondyloarthritis, IBD = Inflammatory Bowel Disease

**Supplementary figure S1** Percentage of women with an immune mediated inflammatory disease diagnosis receiving ≥1 prescription for an NSAID, corticosteroid or csDMARD during the year before, during or year after pregnancy

Rheumatoid arthritis

Percentage of pregnancies

| Time period | Azathioprine | Balsalazide | Hydroxychloroquine | Ciclosporin | Leflunamide | Mercaptopurine | Mesalamine | Methotrexate | Olsalazine | Penicillimine | Sulfasalazine |
| --- | --- | --- | --- | --- | --- | --- | --- | --- | --- | --- | --- |
| Pre 4 | 4.08163 | 0 | 34.949 | 1.53061 | 2.29592 | 0 | 0 | 38.0102 | 0 | 0 | 44.1327 |
| Pre 3 | 4.39276 | 0 | 34.3669 | 1.55039 | 2.06718 | 0 | 0 | 31.0078 | 0 | 0.258398 | 48.8372 |
| Pre 2 | 4.43213 | 0 | 36.5651 | 1.66205 | 2.77008 | 0 | 0 | 24.3767 | 0 | 0 | 50.9695 |
| Pre 1 | 6.55271 | 0 | 34.4729 | 1.4245 | 2.5641 | 0 | 0.2849 | 17.3789 | 0 | 0 | 54.1311 |
| Tri 1 | 7.0922 | 0 | 36.1702 | 1.06383 | 2.48227 | 0 | 0 | 13.1206 | 0 | 0 | 55.6738 |
| Tri 2 | 10.7692 | 0 | 36.9231 | 1.53846 | 0.769231 | 0 | 0 | 2.30769 | 0 | 0 | 61.5385 |
| Tri 3 | 8.77193 | 0 | 38.5965 | 1.75439 | 0 | 0 | 0 | 7.01754 | 0 | 0 | 57.0175 |
| Post 1 | 5.71429 | 0 | 29.8413 | 1.5873 | 3.1746 | 0 | 0 | 30.4762 | 0 | 0 | 46.0317 |
| Post 2 | 5.76923 | 0 | 28.022 | 1.37363 | 4.12088 | 0 | 0 | 37.9121 | 0 | 0 | 44.2308 |
| Post 3 | 5.58376 | 0 | 29.1878 | 1.26904 | 3.29949 | 0 | 0 | 40.6091 | 0.253807 | 0 | 45.1777 |
| Post 4 | 4.62287 | 0 | 29.4404 | 0.973236 | 2.91971 | 0 | 0 | 42.8224 | 0.243309 | 0 | 44.5255 |

Psoriatic arthritis

| Time period | Azathioprine | Balsalazide | Hydroxychloroquine | Ciclosporin | Leflunamide | Mercaptopurine | Mesalazine | Methotrexate | Olsalazine | Penicillimine | Sulfasalazine |
| --- | --- | --- | --- | --- | --- | --- | --- | --- | --- | --- | --- |
| Pre 4 | 1.76991 | 0 | 10.6195 | 2.65487 | 7.9646 | 0 | 0 | 38.9381 | 0 | 0 | 46.0177 |
| Pre 3 | 3.50877 | 0 | 10.5263 | 2.63158 | 7.89474 | 0 | 0 | 36.8421 | 0 | 0 | 47.3684 |
| Pre 2 | 3.92157 | 0 | 8.82353 | 2.94118 | 9.80392 | 0 | 0 | 29.4118 | 0 | 0 | 50.9804 |
| Pre 1 | 3.7037 | 0 | 10.1852 | 5.55556 | 9.25926 | 0 | 0 | 24.0741 | 0 | 0 | 55.5556 |
| Tri 1 | 7.24638 | 0 | 13.0435 | 7.24638 | 7.24638 | 0 | 0 | 23.1884 | 0 | 0 | 46.3768 |
| Tri 2 | 15.3846 | 0 | 7.69231 | 7.69231 | 3.84615 | 0 | 0 | 11.5385 | 0 | 0 | 57.6923 |
| Tri 3 | 18.1818 | 0 | 9.09091 | 4.54545 | 0 | 0 | 0 | 13.6364 | 0 | 0 | 54.5455 |
| Post 1 | 2.5 | 0 | 11.25 | 2.5 | 7.5 | 0 | 0 | 35 | 0 | 0 | 46.25 |
| Post 2 | 3.63636 | 0 | 7.27273 | 2.72727 | 4.54545 | 0 | 0 | 45.4545 | 0 | 0 | 42.7273 |
| Post 3 | 2.41935 | 0 | 6.45161 | 3.22581 | 3.22581 | 0 | 0 | 50 | 0 | 0 | 41.9355 |
| Post 4 | 2.27273 | 0 | 6.06061 | 5.30303 | 3.78788 | 0 | 0 | 46.2121 | 0 | 0 | 44.697 |

Psoriasis

| Time period | Azathioprine | Balsalaz | Hydroxychloroquin | Ciclosporin | Leflunamide | Mercap | Mesalazine | Methotrexate | Olsalazine | Penicilliman | Sulfasalazine |
| --- | --- | --- | --- | --- | --- | --- | --- | --- | --- | --- | --- |
| Pre 4 | 1.38889 | 0 | 20.8333 | 31.9444 | 1.38889 | 0 | 5.55556 | 33.3333 | 0 | 0 | 6.94444 |
| Pre 3 | 1.49254 | 0 | 17.9104 | 34.3284 | 1.49254 | 0 | 7.46269 | 32.8358 | 0 | 0 | 7.46269 |
| Pre 2 | 1.42857 | 0 | 22.8571 | 30 | 0 | 0 | 8.57143 | 31.4286 | 0 | 0 | 8.57143 |
| Pre 1 | 3.57143 | 0 | 21.4286 | 37.5 | 0 | 0 | 7.14286 | 21.4286 | 0 | 0 | 14.2857 |
| Tri 1 | 5.12821 | 2.5641 | 30.7692 | 30.7692 | 2.5641 | 0 | 10.2564 | 12.8205 | 0 | 0 | 10.2564 |
| Tri 2 | 15.3846 | 0 | 30.7692 | 15.3846 | 0 | 0 | 23.0769 | 0 | 0 | 0 | 30.7692 |
| Tri 3 | 8.33333 | 0 | 25 | 16.6667 | 0 | 0 | 25 | 0 | 0 | 0 | 25 |
| Post 1 | 1.85185 | 1.85185 | 16.6667 | 33.3333 | 1.85185 | 0 | 9.25926 | 25.9259 | 0 | 0 | 12.963 |
| Post 2 | 2.63158 | 0 | 17.1053 | 21.0526 | 0 | 0 | 7.89474 | 35.5263 | 0 | 0 | 18.4211 |
| Post 3 | 3 | 1 | 13 | 14 | 1 | 0 | 12 | 46 | 0 | 0 | 14 |
| Post 4 | 3.0303 | 0.757576 | 13.6364 | 12.1212 | 1.51515 | 0 | 9.84848 | 48.4848 | 0 | 0 | 14.3939 |

Spondyloarthritis

Percentage of pregnancies

| Time period | Azathioprine | Balsalazide | Hydroxychloroquine | Ciclosporin | Leflunamide | Mercaptopurine | Mesalamine | Methotrexate | Olsalazine | Penicillimine | Sulfasalazine |
| --- | --- | --- | --- | --- | --- | --- | --- | --- | --- | --- | --- |
| Pre 4 | 0 | 0 | 14.2857 | 0 | 0 | 0 | 0 | 9.52381 | 0 | 0 | 80.9524 |
| Pre 3 | 0 | 0 | 13.0435 | 0 | 0 | 0 | 0 | 8.69565 | 0 | 0 | 82.6087 |
| Pre 2 | 0 | 0 | 17.3913 | 0 | 0 | 0 | 0 | 8.69565 | 0 | 0 | 73.913 |
| Pre 1 | 5.26316 | 0 | 10.5263 | 0 | 0 | 0 | 0 | 0 | 0 | 0 | 84.2105 |
| Tri 1 | 0 | 0 | 21.4286 | 0 | 0 | 0 | 0 | 0 | 0 | 0 | 78.5714 |
| Tri 2 | 0 | 0 | 40 | 0 | 0 | 0 | 0 | 0 | 0 | 0 | 60 |
| Tri 3 | 0 | 0 | 40 | 0 | 0 | 0 | 0 | 0 | 0 | 0 | 60 |
| Post 1 | 0 | 0 | 11.7647 | 0 | 0 | 0 | 0 | 5.88235 | 0 | 0 | 82.3529 |
| Post 2 | 0 | 0 | 12.5 | 0 | 0 | 0 | 0 | 6.25 | 0 | 0 | 81.25 |
| Post 3 | 4.16667 | 0 | 8.33333 | 0 | 0 | 0 | 0 | 12.5 | 0 | 0 | 75 |
| Post 4 | 3.7037 | 0 | 11.1111 | 0 | 0 | 0 | 0 | 7.40741 | 0 | 0 | 81.4815 |

Inflammatory bowel disease

| Time period | Azathioprine | Balsalazide | Hydroxychloroquine | Ciclosporin | Leflunamide | Mercaptopurine | Mesalamine | Methotrexate | Olsalazine | Penicillimine | Sulfasalazine |
| --- | --- | --- | --- | --- | --- | --- | --- | --- | --- | --- | --- |
| Pre 4 | 32.5328 | 4.25764 | 0.327511 | 0.163755 | 0 | 4.4214 | 72.2707 | 0.491266 | 0.982533 | 0 | 3.71179 |
| Pre 3 | 30.3377 | 4.08497 | 0.217865 | 0.217865 | 0 | 4.62963 | 73.2026 | 0.43573 | 1.19826 | 0 | 3.15904 |
| Pre 2 | 30.9549 | 4.35467 | 0.262329 | 0.262329 | 0 | 4.14481 | 73.8195 | 0.367261 | 1.10178 | 0 | 2.67576 |
| Pre 1 | 29.9517 | 3.97209 | 0.322061 | 0.161031 | 0 | 3.97209 | 74.1278 | 0.268384 | 1.01986 | 0 | 2.57649 |
| Tri 1 | 30.0756 | 3.66492 | 0.116347 | 0.116347 | 0 | 4.01396 | 74.9273 | 0.0581734 | 0.8726 | 0 | 2.44328 |
| Tri 2 | 28.6237 | 3.29429 | 0.0732064 | 0.146413 | 0 | 3.73353 | 75.4026 | 0.146413 | 0.805271 | 0 | 2.34261 |
| Tri 3 | 28.5481 | 3.01794 | 0 | 0.163132 | 0 | 3.99674 | 74.5514 | 0.0815661 | 0.897227 | 0 | 2.28385 |
| Post 1 | 29.6645 | 3.64921 | 0.117716 | 0.176574 | 0 | 4.00235 | 73.9847 | 0.176574 | 0.824014 | 0 | 1.94232 |
| Post 2 | 30.1471 | 3.95928 | 0.169683 | 0.226244 | 0 | 4.24208 | 73.5294 | 0.339367 | 0.961538 | 0 | 2.20588 |
| Post 3 | 31.1791 | 4.19501 | 0.226757 | 0.226757 | 0 | 4.42177 | 72.1655 | 0.283447 | 0.907029 | 0 | 2.32426 |
| Post 4 | 31.9294 | 3.81332 | 0.284576 | 0.170746 | 0 | 4.78088 | 72.3961 | 0.398406 | 0.739898 | 0 | 2.44735 |

**Supplementary figure S2** Percentage of women receiving ≥1 prescription for a csDMARD during each of the 3-month time periods by product

Percentages are shown separately for each IMID and for each 3-month time period in the year before, during and year after pregnancy. Women may have been receiving a prescription for more than one csDMARD during a single 3-month time period and therefore some percentages total more than 100.
